# *Tropilaelaps mercedesae* parasitism changes behavior and gene expression in honey bee workers

**DOI:** 10.1371/journal.ppat.1009684

**Published:** 2021-07-08

**Authors:** Jing Gao, Shilong Ma, Xinling Wang, Yang Yang, Qihua Luo, Xing Wang, Feng Liu, Qiang Wang, Zhongmin Fu, Qingyun Diao, Pingli Dai

**Affiliations:** 1 Key Laboratory of Pollinating Insect Biology, Institute of Apicultural Research, Chinese Academy of Agricultural Sciences, Beijing, China; 2 College of Bee Science, Fujian Agriculture and Forestry University, Fuzhou, China; 3 Miyun Apicultural Station, Beijing, China; 4 Beijing Apicultural Station, Beijing, China; 5 Jiangxi Institute of Apicultural Research, Nanchang, China; University of Illinois at Urbana-Champaign, UNITED STATES

## Abstract

*Tropilaelaps mercedesae* is one of the most problematic honey bee parasites and has become more threatening to the beekeeping industry. *Tropilaelaps* can easily parasitize immature honey bees (larvae and pupae) and have both lethal and sublethal effects on the individual worker bees. Our study for the first time experimentally assessed the effects of *T*. *mercedesae* on olfactory learning, flight ability, homing ability as well as transcriptional changes in parasitized adult honey bees. *T*. *mercedesae* infestation had negative impacts on olfactory associated function, flight ability, and homing rate. The volume of the mushroom body significantly increased in infested honey bees, which may be correlated to the lower sucrose responsiveness as well as lower learning ability in the infested bees. The gene expression involved in immune systems and carbohydrate transport and metabolism were significantly different between infested bees and non-infested bees. Moreover, genes function in cell adhesion play an essential role in olfactory sensory in honey bees. Our findings provide a comprehensive understanding of European honey bees in response to *T*. *mercedesae* infestation, and could be used to further investigate the complex molecular mechanisms in honey bees under parasitic stress.

## Author summary

In recent decades, there has been serious concern about the decline of honey bees in the world. One of the most serious factors contributing to bee population declines is mite parasitism. Although *Varroa destructor* is the most widespread globally, *Tropilaelaps mercedesae* displays greater threat to bee colonies due to its smaller size, shorter phoretic phase, more rapid locomotion, as well as faster reproductive rate. *Tropilaelaps* mites, originally parasite of the giant Asian honey bees, now becoming an emerging threat of European honey bees (*Apis mellifera*) in Asian area. This work aimed to investigate the influence of *T*. *mercedesae* infestation on behavior and gene expression in *A*. *mellifera*. Our results highlight the *T*. *mercedesae* infestation induced negative effects of olfactory learning, flight ability, homing ability of honey bee workers. Moreover, we found that *T*. *mercedesae* infestation caused the up-regulation of genes involved in immune systems and carbohydrate mechanism which were correlated to the different olfactory learning performance in infested honeybee. In addition, genes function in cell adhesion play an essential role in olfactory sensory in honey bees. Our results increase the knowledge of proximate mechanisms in honey bee responding to parasitic stress.

## Introduction

Pollinators, especially genus *Apis*, are critical for the production of agricultural crops and maintaining ecosystems. In recent decades, honeybees have consistently declined in population and biodiversity, posing a potential threat to the existence of species and global food security [1]. One of the most critical factors contributing to bee population declines is parasite infestation, which has caused thousands of colony losses worldwide [2]. Previous studies have demonstrated that parasitic mites in the genus *Varroa* and *Tropilaelaps* are the major factors causing the collapse of European honey bee (*Apis mellifera*) in Asian area [3–6].

*Tropilaelaps* mites are originally parasites of giant Asian honeybees, and divided into four species (*T*. *clareae*, *T*. *koenigerum*, *T*. *mercedesae* and *T*. *thaii*) according to genetic and morphological variation [7]. Over the past few decades, scientists mainly focused on the *Varroa* mite as it is the worst bee pest worldwide, while the *Tropilaelaps* mites are only found in Asia. It has been suggested that *T*. *clareae* and *T*. *mercedesae* have successfully transferred to *A*. *mellifera*, whereas *T*. *mercedesa* has a wider distribution than *T*. *clareae* [6]. Compared with *Varroa*, *Tropilaelaps* mites have smaller size, shorter phoretic phase, more rapid locomotion, as well as faster reproductive rate [8–10]. With these characteristics, *Tropilaelaps* mites present a greater threat than the infamous *Varroa destructor* to *A*. *mellifera*. Until now, there is no report concerning the *Tropilaelaps* mites found in European apiculture industry. With the frequent transfer of commercial bee colonies, the bee trade globalization, the natural proliferation of bees, and the gradual warming of the global climate, it may be only a matter of time until *Tropilaelaps* spread outside of Asia to cause devastating effects on apiculture industry in Europe and North America [6].

*T*. *mercedesae* infestation threatens to the health of *A*. *mellifera* in many aspects. Similar to *Varroa*, *Tropilaelaps* mite can reproduce in the drone and worker brood cells of *A*. *mellifera*, but exclusively feed on brood due to the morphology of their mouthparts and body shape [11]. The life span of *T*. *mercedesae* on pupae and larvae is longer than that on adult bees, indeed, the mites can only survive for two or three days when there are only adult bees around [5,12]. This characteristic makes their dispersal ability not as strong as the *Varroa* mites, as they can only disperse on adult bees. Previous studies have indicated that the multiple small wounds derived from feeding of *T*. *mercedesae* cause irregular brood patterns, malformed honey bees that are wingless, and dead larvae [6,9]. Like *Varroa* mites, *Tropilaelaps* are potential vectors of honey bee viruses, particularly Deformed Wing Virus (DWV) [13]. Honey bee infested by *Tropilaelaps* during early development stage may enhance viral proliferation in colony, as the longer exposure to the virus and the stress on susceptibility to viral infection [14]. de Guzman et al. has demonstrated that bee pupae infested with either *Varroa* or *Tropilaelaps* had higher levels of both DWV variants than uninfested pupae [15]. In addition, feeding by *Tropilaelaps* mites can induce cellular immune response in worker broods, which may be caused by the injury from mite feeding, viruses infection, or the interactions of both factors [16]. Although lacking a phoretic phase involving honey bee adults, the adverse effects of *Tropilaelaps* infestation during the precapping stages may last to the adult stage, with remarkably higher numbers of wounds and virus infection rates observed in infested adult honey bees [17]. Infestation of *T*. *mercedesae* lead to a reduction in weight and longevity, and increase of the sugar syrup and pollen consumption in adult worker bees [4,18]. The combination of *T*. *mercedesae* infestation and imidacloprid 50 μg/L exposure reduced survival and increased pollen consumption of *A*. *mellifera* [19].

Despite the various lethal and sublethal effects of *T*. *mercedesae* on *A*. *mellifera*, information about the effects of *T*. *mercedesae* infestation on behavior, organization and molecular mechanisms in individual honey bees is rare [9]. It has been demonstrated that *Varroa* infestation changed the in-hive behavior of adult honeybees during the pupal stage, such as less involved in tending larvae and dealing with hive duties. *Varroa*-infested foraging bees and drones show reduced flight and homing ability [20–22]. Moreover, *Varroa* parasitization has a negative impact on the cognitive abilities of adult bees, which may be associated with DWV boosted by *V*. *destructor* infestation [23,24]. In addition to its effects on behavior performance, *Varroa* infection also causes changes in gene expression related to energetic metabolism and immune response [25]. Transcript levels of pathogen recognition gene Peptidoglycan Recognition Proteins (PGRPs) and Prophenoloxidases (PPOs), key enzymes of the melanization reaction and defense mechanism of insects, increased in honey bee larvae exposed to *Varroa* mites [26]. Comparing the transcriptome changes between *A*. *mellifera* and *A*. *cerana* inducing by *Varroa* also shows that the transcriptional expression changes of components responsible for neuronal rewiring, olfaction, metabolism and social behavior may be the key components driving *Varroa* tolerance [27]. Whether the the negative effects of *Varroa* on honey bees can be inferred to *T*. *mercedesae* keeps unknown.

In this study, we comprehensively investigated the effects of *T*. *mercedesae* infestation on a series of behaviors performance, including sucrose responsiveness, olfactory learning, flight and homing ability. Here we specifically tested the hypothesis that the *T*. *mercedesae* infestation was correlated with the sensory perception of smell and olfactory learning and memory in the honey bees which can be reflected by changes in the expression of specific genes. This work will contribute to a better understanding of alterations in the physiological and molecular traits in response to *T*. *mercedesae* infestation in adult honey bees.

## Materials and methods

### Honey bees and mites

*A*. *mellifera* and *T*. *mercedesae* were obtained from an apiary at the Institute of Apicultural Research (40°00’28”N, 116°12’18”E), Chinese Academy of Agricultural Sciences (Beijing, China), during June to October 2018. In the present study, we used ten healthy and strong colonies with no history of bee diseases, five to the *T*. *mercedesae* treatment and five to the reproduction of the *T*. *mercedesae*. Each colony consisted of six or seven frames of adult bees and two or three frames of brood, which are established in a standard 10-frame Langstroth hive. Since *T*. *mercedesae* can only survive for two or three days when there are only adult bees around, we caged queens for one month and removed all brood from the experimental colonies before the experiments to obtain healthy and mite-free colonies. In each colony, broods at the same age were obtained by caging the queen on an empty frame for 24 h to lay eggs. The comb with eggs was divided into small pieces and fixed on a small frame to constitute special comb, and then put back into the original colony [19].

The reproduction of the *T*. *mercedesae* in *A*. *mellifera* colonies was according to the method reported by Khongphinitbunjong et al [28]. In short, one foundress of *Tropilaelaps* was introduced into a brood cell with a 5th larval instar after sealing, and mites were inoculated into newly sealed brood cells [19]. All inoculum mites were randomly obtained from highly infested *A*. *mellifera* colonies. Other combs with open and closed brood cells without mites were used as controls.

A total of 10 brood combs, five infested combs and five controls were placed in an incubator (34 ± 1°C, 60 ± 10% relative humidity, and darkness). To avoid contamination of the control bees by the infested frame, the control and infested brood frames were incubated in the same incubator but in different layers. Newly emerged bees that infested or non-infested *T*. *mercedesae* were collected every 2 h and randomly placed in cages (9×9×10 cm) with 30 bees as a group. The bees were supplied with sufficient syrup (50% w/v sucrose solution) and fresh pollen, and the cages were maintained in the dark in an incubator (30 ± 1°C, RH 60 ± 10%) [29].

### Flight ability

The flight ability of infested and non-infested worker bees was tested at 15th day post emergence by using a modified flight mill [30]. This procedure was performed according to the flight procedure reported by Tosi *et al*.[31]. On each trial, fifteen honey bees were captured from each comb as one replicate. Honey bees were attached to the wire flight mill arm through a 1-cm-long hollow Teflon tube (S1 Fig). Once the honey bees were attached to the flight mill, a computer connected to the flight mill sensor could record mean velocity, flight duration, and flight distance. The experiment was carried out in three replicates and 45 bees for each replicate were employed with 24 h test duration.

### Homing ability

Every 2 h, 300 infested and non-infested bees were randomly collected from brood combs of three colonies, respectively. Bees were marked with different color painted on their thorax and placed into three non-infested colonies. To familiarize the tested bees with the experimental background, the experiment started three days after collection from the colonies and introduction into the new colony. Then bees were placed in an incubator (26°C± 1°C, 60 ± 10% relative humidity, darkness) for 2 h. All bees were then released from approximate 50 m away from the hive and each bee was only released once. The homing time and the number of honey bees successfully returning to the hive were recorded.

### Sucrose responsiveness and olfactory learning behavior

Sucrose responsiveness was tested according to the standard method for honey bees described in Decourtye et al with with minor changes [32]. Honey bees for proboscis extension reflex (PER) were secured individually in 1.5 mL tubes with their antennae and mouthparts free (S2 Fig). Before feeding assay, tested bees were starved for 4 h, and then keep them in the incubator between testing. Infested and non-infested bees were tested for their response to 30% (w/w) sucrose solutions at 0 (emergence within 24 hours), 5, 10 and 15 days after emergence.

Sucrose stimulation was performed with a soaked toothpick touching both of the bee’s antennae at the same time for 1 s, and the PER was recorded (1 if a bee extended her proboscis and 0 if she did not respond). Alternated water trials between each sugar solution were used to reduce the possible effect of sensory sensitization to antennal touch. All stimulations were performed at 3 min intervals. To avoid invalid counts caused by bees who are only thirsty or not responding to sucrose, bees that responded to water or did not respond to any test concentrations of sugar solution were discarded before the test. The test bees were put back to the incubator after tests were completed. The number of bees died during the experiment was very small and was not statistically analyzed.

Honey bees showing PER after stimulated with a 30% sucrose solution at 15 day after emergence were selected for the olfactory learning behavior test according to Strube-Bloss et al [33]. In short, Linalool (Sigma, 97% purity) acted as the conditioned stimulus through airflow system. In order to familiarize with the mechanical stimulation and with the experimental background, bees were placed in the airflow system with main airflow speed of 50 ml ∙ s-1 added to a secondary airflow speed of 2.5 ml ∙ s-1. The Linalool was soaked on a filter paper strip inserted in a Pasteur pipette cartridge and then delivered through the secondary flow (2.5 ml ∙ s-1) for 6 s. Bees were starved for 4 h prior to conditioning. Three conditioning sessions for individuals were performed at 20 min intervals (conditioning phases C1, C2 and C3). The individuals were then subjected to five test trials (called T1–T5), during which the conditioned stimulus was delivered for 6 s at 20 min intervals without an unconditioned stimulus or a reward with a sucrose solution. The conditioned PER was recorded as a yes-or-no response during the test trials. In each experimental group, 90 bees were conditioned. The experiments were replicated at least three times. After the tests, the bees with different performance were separated and frozen with liquid nitrogen for RNA-seq analysis.

### Scanning electron microscopy of the antennae

Infected and non-infected worker bees were randomly collected from each cage on 0, 5, 10 and 15 day after emergence. The 4^th^, 7^th^, and 8^th^ flagella of the right antennae were observed according to the method reported by Letzkus et al. with scanning electron microscopy [34].

### Paraffin-embedded brain tissue sections

Paraffin-embedded tissue sections of the brains were made according to a previously described method [35]. The test bees were randomly selected from different control and infested colonies. Brain tissue of honey bees at 15th day after emergence were dissected from the head capsule and fixed with 2.5% glutaral. The hematoxylin- and eosin-stained ultrathin sections were observed with transmission electron microscopy.

### Experiment design for RNA-seq analysis

We present an experiment designed to test the molecular effects of *T*. *mercedesae* Infestation on the irregular performance associated with olfactory function observed in the behavior experiment described above. We collected heads from parasitized or non-parasitized bees with different sucrose responsiveness and olfactory learning behavior for RNA-seq sequencing: (1) non-mite-infested honey bees non-extended the proboscis with 30% sucrose stimulus (CKSN); (2) honey bees infested with T. mercedesae and non-extended the proboscis with 30% sucrose stimulus (TSN); (3) non-mite-infested honey bees non-extended the proboscis with odour stimulus in the first test trial (CKN); (4) honey bees infested with T. mercedesae and non-extended the proboscis by touching the antennae with odour stimulus in the first test trial (TN); (5) non-mite-infested honey bees extended the proboscis during five test trial (CKL); (6) honey bees infested with T. mercedesae and extended the proboscis during five test trial (TL). Samples were immediately frozen in liquid nitrogen and stored at -80°C until RNA extraction.

### RNA extraction and RNA sequencing assay

The heads of frozen bees were removed by using a scalpel. Samples were frozen and stored at −80°C until the time of RNA isolation. Total RNA of five bee heads were pooled in each replicate and isolated using TRIzol reagent following the manufacturer’s instruction. For each experimental group, three biological replicates were isolated. A total amount of 3 μg RNA per sample was used as input material for the RNA sample preparations. Total RNA of each sample was isolated using a Quick RNA isolation kit (Bioteke Corporation, Beijing, China) and then assessed using the RNA Nano 6000 Assay Kit of the Agilent Bioanalyzer 2100 system (Agilent Technologies, CA, USA). The construction of the libraries and the RNA-Seq were performed by the Biomarker Biotechnology Corporation (Beijing, China). The mRNA-Seq libraries were generated using the RNA Library Prep Kit (Illumina Inc., San Diego, CA, USA) following standard Illumina protocols. Second strand cDNA synthesis was subsequently performed using DNA Polymerase I and RNase H. cDNAs were used for PCR amplication. PCR products were purified with AMPure XP beads. The cDNA library was quality assessed on the Agilent Bioanalyzer 2100 system. The mRNA-Seq library was performed on the platform (Illumina Inc., San Diego, CA, USA) following the standard Illumina preparation protocol.

### Bioinformatics analysis of RNA-seq data

Trimmomatic software (Bolger et al., 2014) was used to remove adaptor sequences, empty reads, short reads (<50 bp), reads with an N-ratio >10%, and low-quality regions [36]. Then clean reads from each sample were mapped to the *A*. *mellifera* genome by Hisat2 tools software with a maximum allowance of 2 nucleotide mismatches. The abundance of unigenes was performed by the fragments per kilobase of transcript per million fragments mapped (FPKM) method [37]. Differentially expressed genes (DEGs) of two groups were implemented by the DESeq2 R package (1.18.0) with the threshold of │log2 (fold change) │≥ 1 and a false discovery rate (FDR) ≤0.01. The resulting *p*-values were adjusted using the Benjamini and Hochberg’s approach for controlling the false discovery rate. For functional prediction, the sequences were compared with the NCBI-nonredundant protein (NR) database, the Swiss-Prot protein database, and Clusters of Orthologous Groups (COG). The functional classification of the DEGs was implemented by the topGO R packages based on a Fisher’s exact test and FDR correction of 0.01, and the statistical enrichment of DEGs in KEGG pathways were performed by KOBAS software against the KEGG (Kyoto Encyclopedia of Genes and Genomes) database (http://www.kegg.jp/).

### Statistical analyses

We used generalized linear models (GLM) with Tukey’s HSD in SAS (Cary, NC; SAS Institute 2000) to determine whether *T*. *mercedesae* parasitism affected flight velocity, flight distance, flight duration, homing time, and proportion of successful homing flights (p < 0.05). Mite parasitism and colony were used as independent factors. To test for any significant effects of colonies and parasitism, we inspected the colony x pathogen interaction term and therefore kept them in all models, independently of whether they were statistically significant or not. The volume of mushroom bodies was tested using one-way ANOVA followed by Tukey’s HSD (p < 0.05). Chi-square test were applied to analyze the proportion of PER by using SPSS. 22. *P* < 0.05 was considered statistically significant.

## Results

### Flight and homing ability

**Fig 1:**
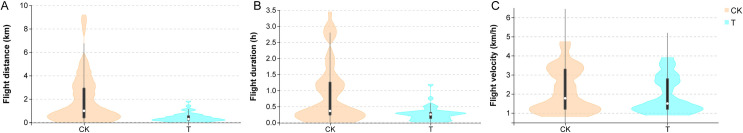
The effects of *T*. *mercedesae* infestation on flight ability of adult honey bee (n = 45). A: flight distance; B: flight duration; C: mean flight velocity. CK: non-infested honey bees; T: *T*. *mercedesae*-infested honey bees. Horizontally, the width of each violin box represents the density of the data values. The white dots represent the median values of each group. The upper and lower edges of the black thick line represent the 3/4 digits and 1/4 digits of the data. The upper and lower ends of the thin line represent the maximum and minimum values of non-outliers of the data.

The flight and home ability of infested and non-infested worker bees was tested on the 15th day post emergence, and each bee flew only once. Control bees flew for 0.84 ± 0.16 h and covered 1.87 ± 0.39 km at an average velocity of 2.18 ± 0.18 km/h. *T*. *mercedesae*-infested bees flew for 0.26 ± 0.05 h and covered 0.44±0.08 km at an average velocity of 1.90 ± 0.20 km/h. Total flight distances (Fig 1A, GLM, colony: *P* = 0.8475, treatment: *P* = 0.0026, colony x treatment: *P* = 0.0102) and flight durations (Fig 1B, GLM, colony: *P* = 0.4861, treatment: *P* = 0.0019, colony x treatment: *P* = 0.0061) was significantly reduced by *T*. *mercedesae* infestation. Neve rtheless, no significant difference of the mean flight velocity was observed between *T*. *mercedesae*-infested worker bees and control bees (Fig 1C, GLM, colony: p = 0.0856, treatment: *P* = 0.4643, colony x treatment: *P* = 0.1705).

**Fig 2:**
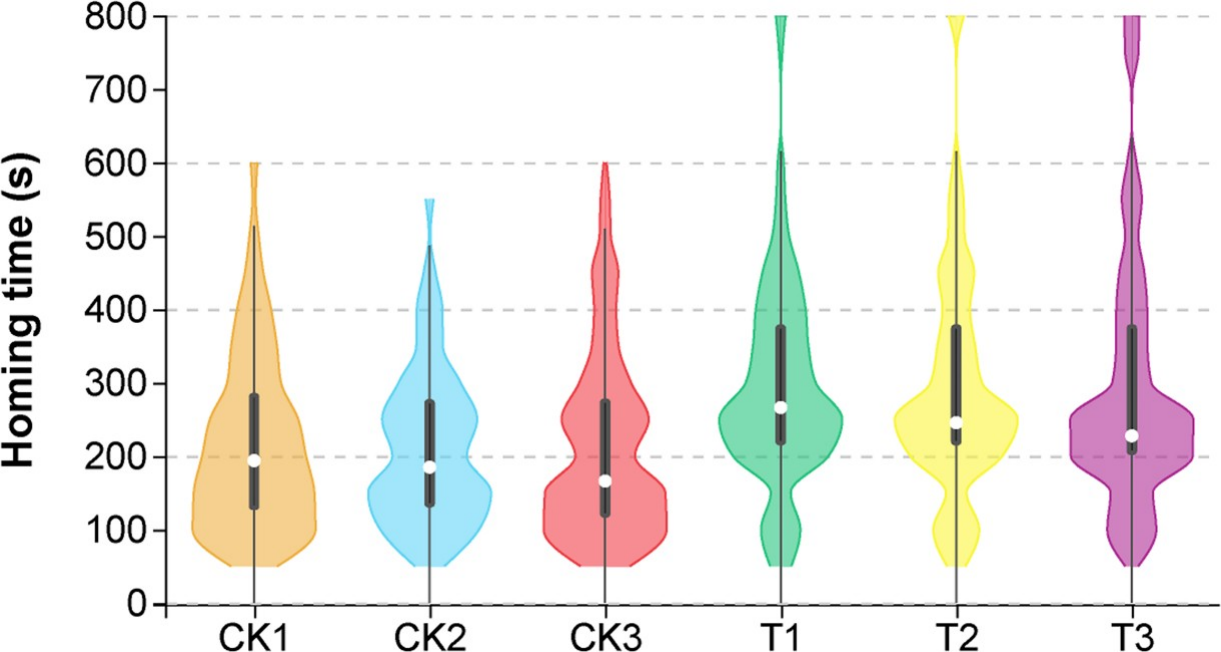
The effect of *T*. *mercedesae* infestation on homing ability of adult honey bee. Violin plot shows the homing time in control bees and *T*. *mercedesae* infested bees (n = 56–96). Horizontally, the width of each violin box represents the density of the data values. The white dots represent the median values of each group. The upper and lower edges of the black thick line represent the 3/4 digits and 1/4 digits of the data. The upper and lower ends of the thin line represent the maximum and minimum values of non-outliers of the data. CK: non-infested honey bees; T: *T*. *mercedesae*-infested honey bees.

The time taken by the infested bees from releasing to arriving at home was 294.53 ± 35.69 s, while that for the non-infested worker bees was 203.29 ± 17.47 s (Fig 2, GLM, colony: *P* = 0.0685, treatment: *P* = 0.0074, colony x treatment: *P* = 0.0083). However, there was no significant difference in the proportion of successful homing flights between the worker bees infested with *T*. *mercedesae* (57.9%) and control bees (70.7%) (χ^2^ = 1.666, *P* = 0.197). In addition, the survival rate of honey bees infested with *T*. *mercedesae* was significantly lower than that of control bees after being put back into the colony (χ^2^ = 4.960, *P* = 0.026, S3 Fig).

### Sucrose responsiveness and olfactory learning performance

**Fig 3:**
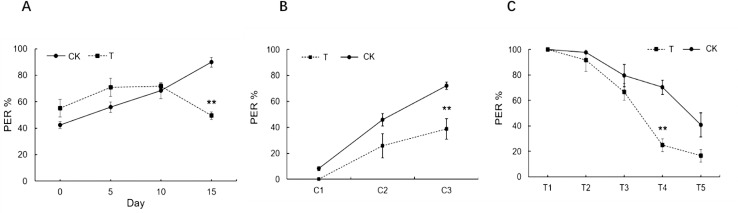
The effect of *T*. *mercedesae* infestation on olfactory associated functions of adult honeybee. A. The effect of *T*. *mercedesae* infestation on sucrose responsiveness of adult honeybee. Infested and control bees were tested for PER to 30% (w/w) sucrose solutions at 0, 5, 10 and 15 days after emergence. PER rate (%) was significantly lower in infested bees at day 15 than that of control group. 0 day indicates newly emerged adult bees. B. PER responses of infested or non-infested honeybees during the conditioning phase (C1–C3). C. PER responses of infested or non-infested honeybees during the extinction phase (T1–T5). CK: non-infested honey bees; T: *T*. *mercedesae*-infested honey bees. Data are means of three independent experiments, and error bars represent ± standard error (SE). Significant differences to CK with P < 0.01 are indicated by asterisks according to chi-squared test.

Next, we investigated the effects of *T*. *mercedesae* on the sucrose responsiveness during worker bee development. As shown in Fig 3A, there was no significant difference in sucrose responsiveness between worker bees infested and non-infested with T. mercedesae on 0 day (χ^2^ = 1.816, P = 0.178), 5th day (χ^2^ = 3.086, P = 0.0790), and 10th day (χ^2^ = 0.195, P = 0.659) after emergence. But on the 15th day, the PER performance of healthy worker bees was significantly higher than that of *T*. *mercedesae*-infested worker bees (χ^2^ = 151.467, *P* < 0.010).

In both control and infested group, the percentage of bees responding to the odor stimuli increased with the number of conditioned trials and decreased with the number of unrewarded trials. In the 1st trial (C1) and the 2nd trial (C2) of the conditioning phase, the olfactory learning performances did not change by *T*. *mercedesae* infestation (*P* > 0.05). At the end of the conditioning period (C3), the PER of infested worker bees was significantly lower than that of non-infested bees (χ^2^ = 9.639, *P* = 0.002, Fig 3B). During testing phase, the responses of control bees decreased from 100% (T1) to approximately 40% (T5), while only about 20% of the infested bees responded to linalool at the end of the testing period (T5). Worker bees infested with *T*. *mercedesae* showed a significantly lower response rate than that of non-infested bees in the 4th trials of the extinction phase (T4) (χ^2^ = 8.167, *P* = 0.004, Fig 3C).

### Illumina sequencing and transcriptome assembly

**Fig 4:**
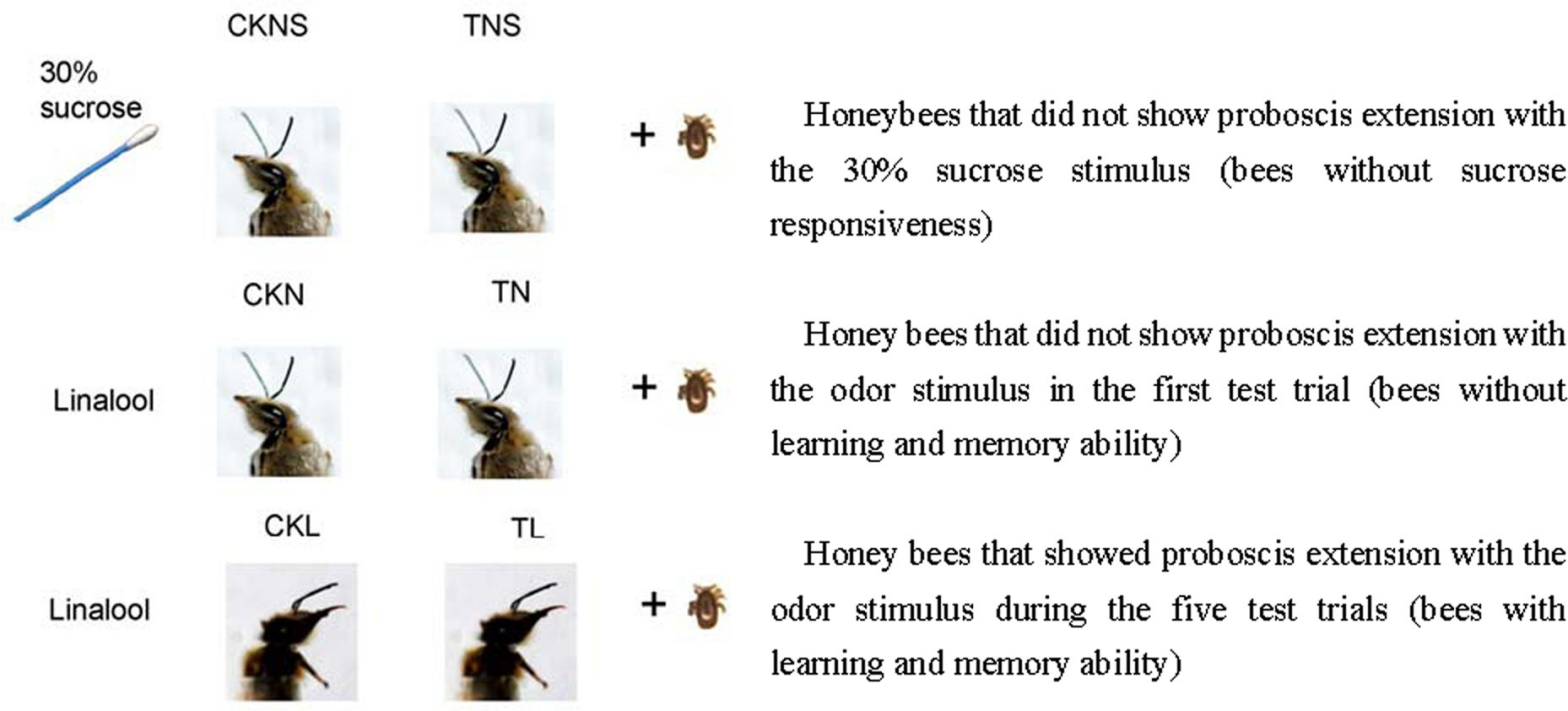
Schematic of RNA-seq experimental design. The experimental design consisted of three groups displaying different sucrose responsiveness or learning ability.

Given the behavioral evidence above, we surmised that *T*. *mercedesae* infestation have a negative impact on the olfactory associated function of honey bees. We therefore analyzed the differences in transcripts between *T*. *mercedesae*-infested (T) and non-infested honey bees (CK) during the olfactory learning process with high-throughput RNA-seq analysis. A graphic overview of the experimental design is shown in Fig 4.

The major sequencing assembly information is summarized in S1 Table. In total of 118 Gb sequencing data of raw reads were obtained from 18 cDNA libraries. After filtering the raw reads, approximately 51.53 million clean reads were generated, with an average GC content of 39.73%. The Q30 in each library was above 92.81%. In total, 10,141 genes (including 1,659 new genes) were identified by sequencing analysis. Over 88% of the genes were shared in all the groups from both *T*. *mercedesae*-infested and non-infested honey bees, representing 74% of the known honeybee genes [38].

### DEGs between T. mercedesae-infested and non-infested honey bees

**Fig 5:**
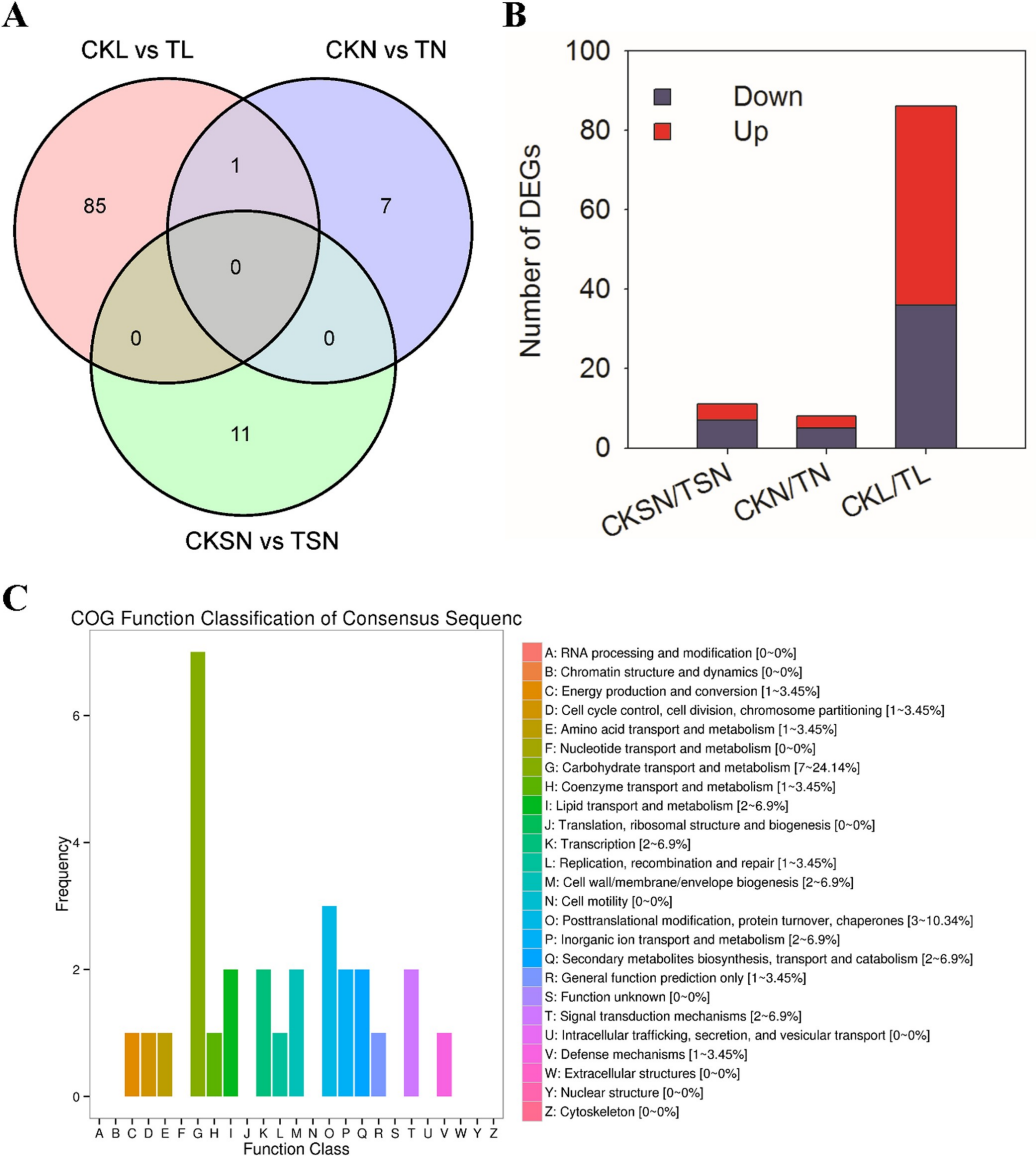
The differentially expressed genes (DEGs) between healthy bees and infested bees. A. The Venn diagram of differentially expressed genes between bees with and without olfactory learning ability in both the *T*. *mercedesae*-treated group and the CK group. B. the number of DEGs in each comparison. Up-regulated and down-regulated means that these genes were higher or lower expressed in infested group compared to CK group. C. COG Function Classification of DEGs in comparision CKL/TL.

A summary of the overall changes in gene expression in various comparison in mite-infested honey bees and non-infested honey bees is shown in S2 Table. Compared with the respective controls, 86, 8, and 11 DEGs were obtained in the *T*. *mercedesae*-infested groups according to different sucrose responsiveness and learning and memory statuses. Only one shared gene, which encodes venom acid phosphatase Acph-1, was observed in the CKN/TN and CKL/TL comparisons (Fig 5A). The pairwise comparisons showed that there were very few differentially expressed genes identified in the comparison of CKN/TN and CKSN/TSN, reflecting bees without PER nor learning and memory ability have similarities in expression patterns in both infected group and control group. We further focused on the transcriptome changes of bees with learning ability between the control group and the infected group (CKL vs. TL). Compared to the CK group, we found that 50 genes were uniquely upregulated, while 36 genes were downregulated in *T*. *mercedesae*-infested bees (Fig 5B). Based on the functional annotation, a total of six unigenes were annotated as being involved in responses to environmental stimulation. Except for NF-kappa-B inhibitor (GB46554), the other five DEGs including genes encoding antimicrobial peptides (GB51223, GB47318, GB47546), potassium voltage-gated channel protein (GB43655) and major royal jelly protein 1 (GB55205) were up-regulated in TL (S2 Table). In addition, a peptidoglycan-recognition protein (GB47805) and a dolichyl-phosphate beta-glucosyltransferase (GB55419) which have been reported positively correlated with *Varroa* sensitive hygiene [39,40], were also upregulated in TL. Then, DEGs were aligned to the COG database to predict and classify their possible functions (Fig 5C). Eight genes were abundant in “Carbohydrate transport and metabolism”. Except for one gene encoding glyceraldehyde-3-phosphate dehydrogenase (GB50901), and other genes were upregulated compared to their levels in the CK group, including genes encoding major royal jelly proteins (GB55205, GB55208, GB55207), alpha-amylase precursor (GB49854), and synaptic vesicle glycoprotein 2C (GB49708) (S3 Table). Neurochondrin is a novel cytoplasmic protein that acts as a negative regulator of Ca^2+^/calmodulin-dependent protein kinase II phosphorylation and is essential for the learning process in mammals [41]. A gene encoding neurochondrin homolog protein (GB45484) was greatly downregulated (28.8-fold) in mite-infested honey bees that showed olfactory learning ability.

### DEGs identified between bees with different sucrose responsiveness statuses

**Fig 6:**
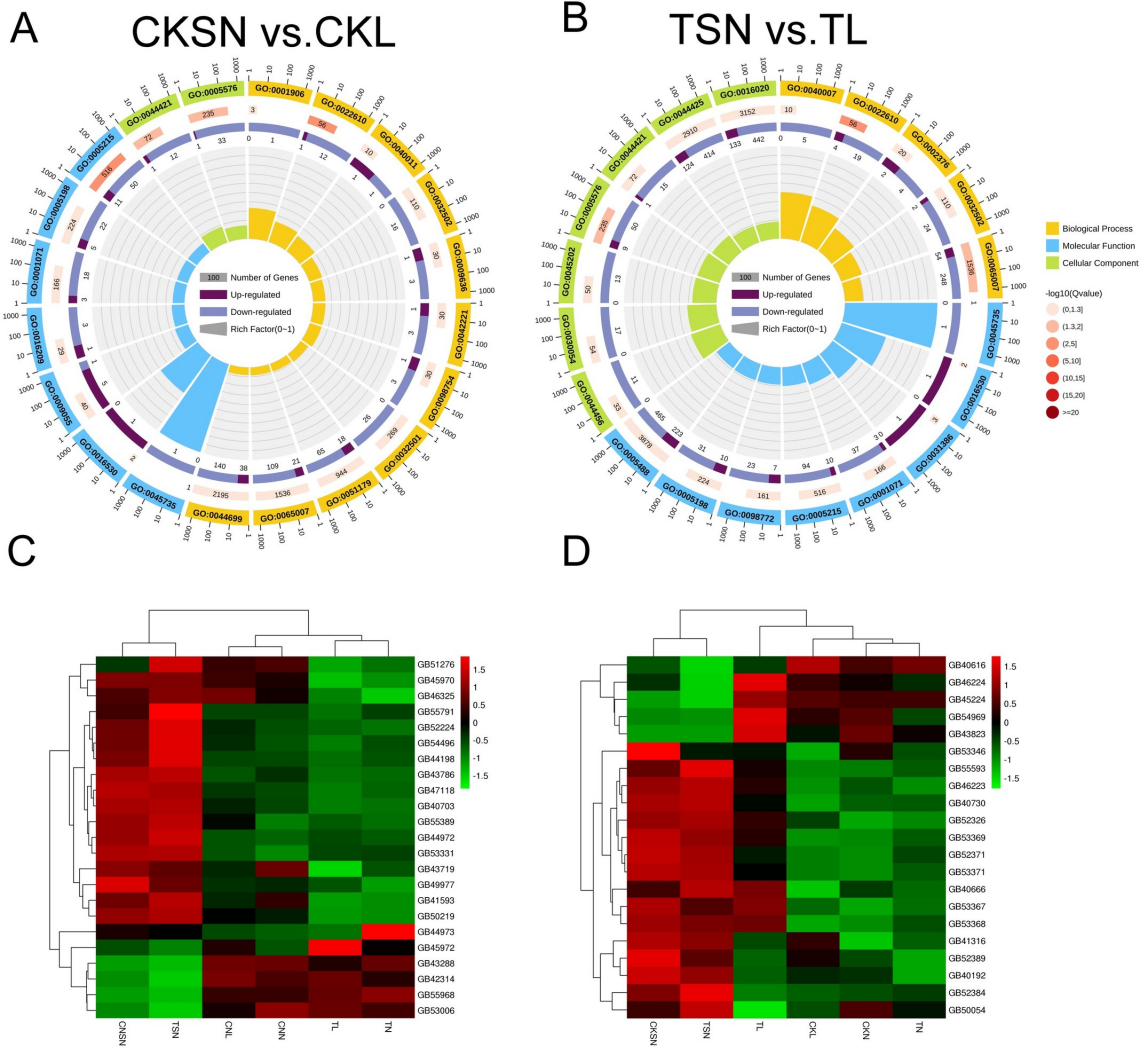
The differentially expressed genes (DEGs) between bees with or without learning ability in CK group and *T*. *mercedesae*-treated group. **A&B**, GO term enrichment analysis in CKSN/CKL and TSN/TL was performed. The first lap indicates top 20 GO term and the number of the genes corresponds to the outer lap. The second lap indicates the number of the genes in the genome background and Q values for enrichment of the DEGs for the specified biological process. The third lap indicates the ratio of the upregulated genes (deep purple) and downregulated genes (light purple). The fourth lap indicates the enrichment factor of each GO term. **C.** Heatmap of related transcripts identified in the comparison between bees with or without learning ability. **D.** Heatmap of chemosensory-related transcripts identified in the comparison between bees with or without learning ability. The colours indicate the log2-transformed expression values, which represent the expression level of a transcript identified in control group or infested group.

We also investigated the gene expression in bees with different sucrose responsiveness statuses in both *T*. *mercedesae*-infested group and CK group. To make the subsequent analysis more concise, CKL and TL were selected as representative groups that bees having sucrose responsiveness given that there were almost no differences in gene expression observed in the comparison of CKL vs. CKN (19 DEGs) and TL vs. TN (11 DEGs). More down-regulated genes were identified in bees with sucrose responsiveness compared to those without sucrose responsiveness, suggesting that bees tend to suppress some related genes instead of activating genes in odorant detection process (S4A Fig). The Venn diagram showed that a large number of DEG in CKSN vs. CKL were also identified in the comparison TSN vs.TL (S4B and S4C Fig). 188 and 510 DEGs were commonly up-regulated or down-regulated among all comparisons, such as genes involved in defense response (e.g. ABC transporters (GB55378, GB55375, GB41616, GB50101)), signal transduction (e.g. Serine/threonine-protein kinase (GB41700, GB53414, GB51427, GB44092, GB48061)), and carbohydrate metabolism (e.g. facilitated trehalose transporters (GB43800, GB41742, GB40972, GB54123, GB47931)). We found 120 and 666 genes were uniquely up-regulated, while 198, and 1003 genes were exclusively down-regulated expressed in each comparison, respectively (S4 Table). The GO term analysis showed that the most abundant terms were highly similar with respect to GO terms in comparison CKSN/CKL and TSN/TL (Fig 6A and 6B). The terms of “binding” (GO:0005488), “catalytic activity” (GO:0003824), “metabolic process” (GO:0008152), and “cell” (GO:0005623) are dominant. While compared to the whole genome background, GO annotation associated with biological adhesion (GO:0022610) was most significantly enriched in both CK and infested group (Q value < 0.05) (S5 and S6 Tables). Interestingly, genes related to cell adhesion, such as neural-cadherin, fat-like cadherin and protocadherin, were all down-regulated in bees with sucrose responsiveness in both CK and infested group (Fig 6C and S8 Table). Additionally, a total of 20 genes involved in sensory system showed clear separation between bees with different sucrose responsiveness statuses, especially odorant binding proteins (OBPs) and odorant receptors (ORs) (Fig 6D and S7 Table). The expression levels of most OBPs and ORs in bees having PER were significantly lower than that in bees without sucrose responsiveness. Particularly, chemosensory protein 1 (GB43823) and an odorant binding protein (GB46224) were exclusively up-regulated in TL groups.

### Morphological alterations in the antenna and mushroom bodies

**Fig 7:**
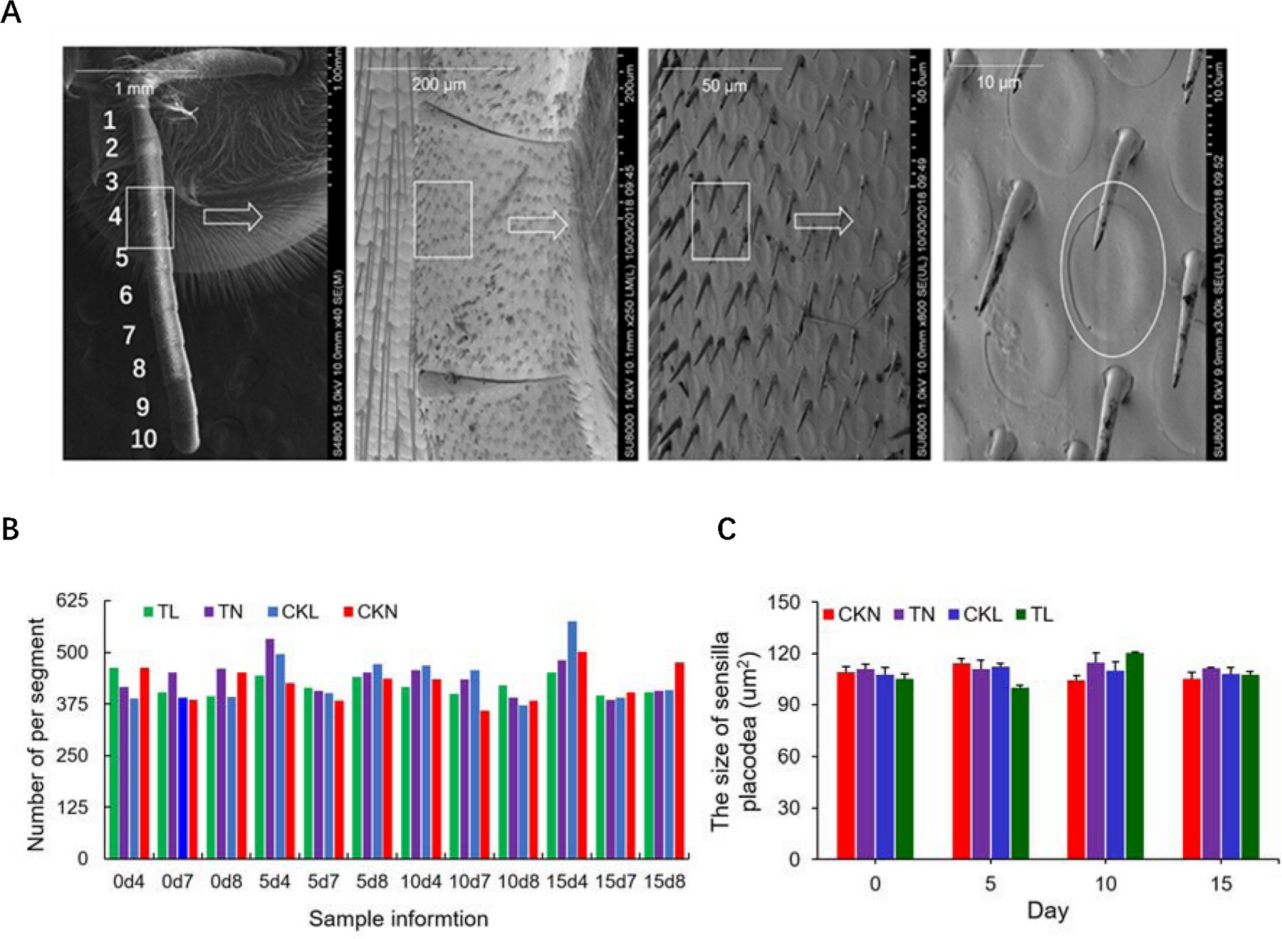
Flagellum of the honey bee antenna observed with scanning electron microscopy (SEM). A. SEM of antennal segments of scape for worker showing antenna and olfactory sensillum B. The number of sensilla placodea on 4, 7, 8 segments in the right antennae of infested and non-infested bees at 0, 5, 10 and 15 days. C. The size of sensilla placodea in the right antennae of infested and non-infested bees 0, 5, 10 and 15 days. For the actual measurement was performed in duplicate (i.e. two biological replicates, A and B) alongside controls.

Further, we investigated the morphological changes of antenna and mushroom bodies, as they are thought to be the most closely organs related to olfactory function in honey bee. By scanning the right antenna of adult honey bee during the development process with scanning electron microscopy, there is no significant differences in the number or size of olfactory cells in the 4th, 7th, and 8th right antennae between T. mercedesae-infested and non-infested bees (Fig 7A–7C).

**Fig 8:**
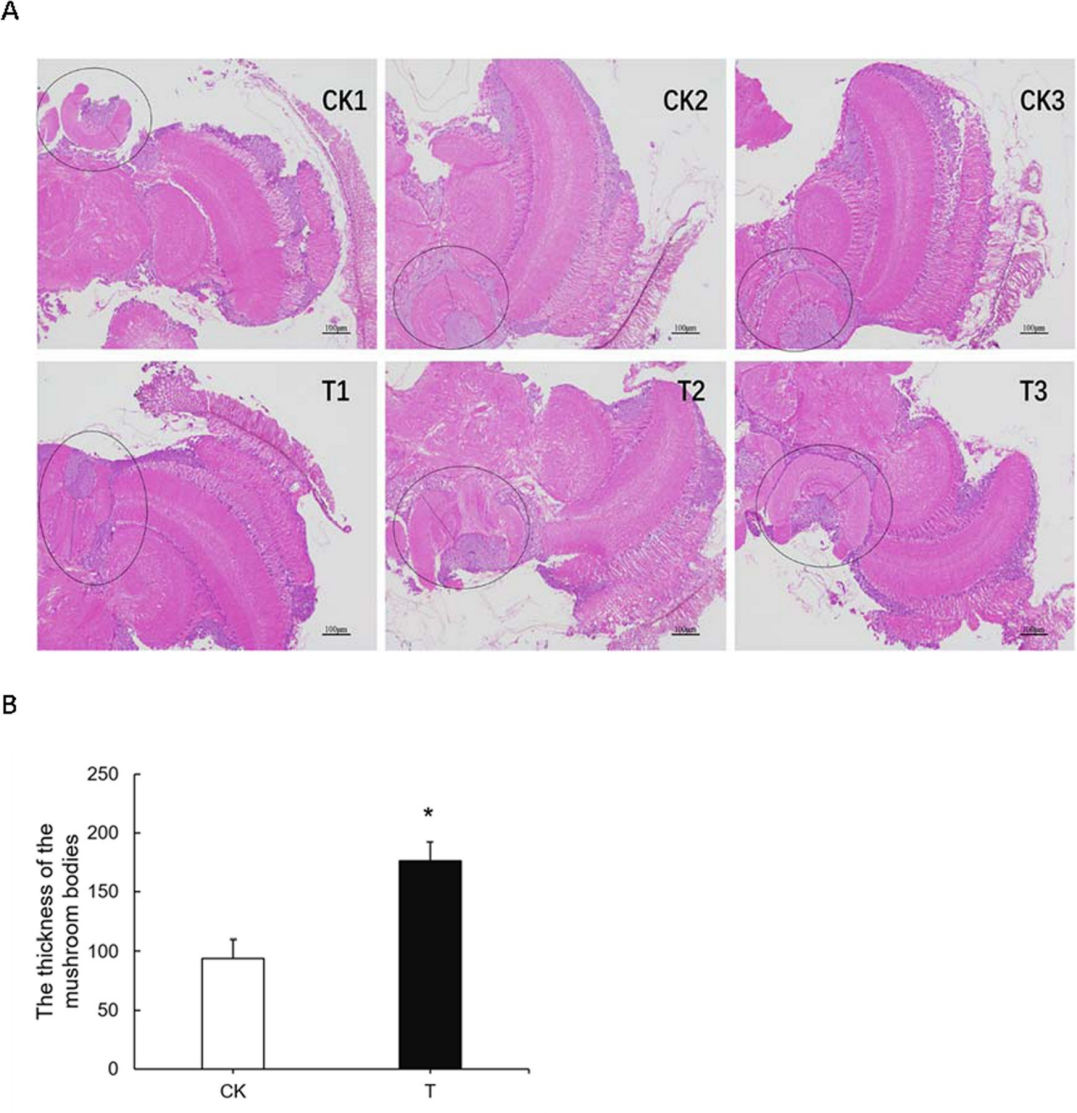
The effect of *T*. *mercedesae* infestation on mushroom body in the brain of adult honey bees. A. Photomicrographs of sections of mushroom body of control worker bees and *T*. *mercedesae* infested bees stained with hematoxylin and eosin. The black circle was indicated mushroom body of individual honeybee. B. The thickness of mushroom bodies of control bees and *T*. *mercedesae* infested bees. Data are means of three biological replicates, and error bars represent ± standard error (SE) (n = 3). Significant differences to CK with P < 0.05 are indicated by an asterisk above the bars and were determined by one-way ANOVA followed by Tukey’s HSD.

Morphological alterations in the mushroom bodies of worker honey bees infested with *T*. *mercedesae* were observed (Fig 8A). The mean thickness of mushroom bodies in *T*. *mercedesae*-infested honey bees was 176.33 ± 15.81 μm, which was significantly thicker than the mushroom bodies in non-infested honey bees (93.89 ± 15.82 μm; F = 13.59, *P* = 0.02; Fig 8B).

## Discussion

In recent years, *T*. *mercedesae* infestation has severely damaged *A*. *mellifera* colonies in China, and these infestations currently threaten the whole beekeeping industry [42]. Compared to *Varroa* destructor, *T*. *mercedesae* is more dangerous to *A*. *mellifera* because of its short life cycle and phoretic stage, which contribute to rapid population development in *A*. *mellifera* colonies [13]. The effects of the parasite might operate within comparatively short time after a mite has entered a bee. Although the the damage caused by *T*. *mercedesae* mites are mainly in pupae and larvae stage, the negative effects may last to adult stages, with remarkably higher numbers of wounds and virus infection observing on adult honey bees [17]. In this study, we found *T*. *mercedesae* infestation negative effected on sucrose responsiveness, olfactory learning, flight and homing ability in worker bees. In addition, we identified genes related to carbohydrate transport and metabolism, stress response of xenobiotics, and neuronal function were differentially expressed between infested bees and healthy bees. Some of these genes have previously been reported as potential predictors of resistance to mite infestation [39]. To our knowledge, this is the first investigation of the molecular mechanism underlying the olfactory dysfunction caused by *T*. *mercedesae* infestation in adult honeybees.

Our results from the flight mills showed that infested bees had a significantly lower flight duration (Fig 1A) and flight distance (Fig 1B) than the control group, but no significant difference was observed between the control and infested bees for the mean velocity (Fig 1C). Previous studies have demonstrated that parasitic *Varroa* mites can impair the mitochondria in flight muscles and negative influence flight duration and homing ability [20,43]. By contrast, honey bees infested with another common bee pathogen, *Nosema ceranae*, displayed a higher flight activity which is correlated to the higher level of ethyl oleate in the parasitized bees [44]. One of the honeybees’ adaptation for flight is dense packing of enzymes for carbohydrate catabolism [45]. We found that eight genes involved in “Carbohydrate transport and metabolism” (GO:) showed significantly different expression between infested and non-infested bees (S3 Table). Glyceraldehyde-3-phosphate dehydrogenase is a key enzyme that catalyzes an important energy-yielding step in carbohydrate metabolism. In this study, the down-regulation of a gene encoding glyceraldehyde-3-phosphate dehydrogenase was observed in the heads of infested bees with learning ability (TL) suggests a lower supply of carbohydrate resources as well as a lower energy supply (Fig 5C). The energy changes may not only occur in the head but also in the whole body of bees. Whether the impairment of the flight ability by *T*. *mercedesae* infestation observed in this study is directly correlated to the identified responsive genes involved in proximate mechanisms underlying the energy supply needs further evidence for validation. The weaken olfactory learning and flying ability can cause a delay in honey bee homing behavior. We found that *T*. *mercedesae* infested-honey bees were significantly reduced in homing proportion and increased homing time versus control group (Fig 2). As with the *Varroa* mites [20] and insecticides [31,46], it appears that the interruption of homing behavior during the foraging process caused by *T*. *mercedesae* infestation will eventually leads to colony failure if bees lose flexibility in their response to colony demands.

The different performance in olfaction may account for the difference in the tolerance of bee to mite parasitism. Bees with higher olfactory sensitivity will initiate hygiene behaviors earlier under the low stimulation intensity of *Varroa* extracts, thus they can accurately detect and eliminate abnormal broods and nestmates [47,48]. In this study, the PER performance to stimulation with 30% sucrose solution of infested bees was significantly lower than that of healthy worker bees (Fig 3A). Additionally, *T*. *mercedesae* infestation depressed olfactory learning and memory ability in honey bees (Fig 3B and 3C). These results are consistent with previous studies on *V*. *destructo*r [49]. It has been demonstrated that *V*. *destructor* parasitization leads to specific impairments in sucrose responsiveness and associative olfactory learning in honeybees, which may be caused by DWV infection boosted by *V*. *destructor* infestation [23].

Along with the behavioral changes induced by *T*. *mercedesae* infestation in honeybees, changes in gene expression related to physiological responses were also observed in the present study. The transcriptional expression patterns of honey bees without sucrose responsiveness (CKSN, TSN) or without learning ability (CKN, TN) were similar between the infested honey and non-infested honey (Fig 4). Several immunity genes showed higher expression level in *T*. *mercedesae*-infested bees (TL) than those in corresponding control group (CKL), including antimicrobial peptides (AMPs) (abaecin precursor (GB47318), apidaecins type 14 precursor (GB47546), hymenoptaecin preproprotein GB51223) and a cytochrome P450 (GB40288) (S2 Table). As key components of the Toll pathway, the AMPs have been regularly reported as responding to parasitic attacks by *Nosema* or *Varroa* [50–52]. The defense response of bees to bacteria occurs through the rapid overexpression of AMPs, which are effectively delivered to the site of infection of natural pathogens and parasites [53,54]. An *in vitro* study showed that *Varroa* parasitism resulted in significantly higher transcript abundance for antimicrobial peptides in developing worker bees [55]. The feeding activity of mites can increase pathogenicity of the virus through immunosuppression of bee host [52,56,57]. Recently, Wu et al. described the tripartite interactions between honey bee pupae, *T*. *mercedesae*, and DWV and demonstrated that the expression levels of Defensin-1 and Hymenoptaecin are induced by DWV replication and *T*. *mercedesae* [58]. They found that the expression of hymenoptaecin in honeybee was negatively correlated with the mite vitellogenin (Vg) gene, which is essential for the reproduction of mite. It is known that neonicotinoid exposure activated NF-κB and subsequently affects the induction of antimicrobial peptides [59]. In this study, a gene encoding an NF-kappa-B inhibitor (GB46554) was found to be down-regulated in *T*. *mercedesae*-infested bees. The positive impact on NF-κB activation reflected the stress responses of honey bees to the parasitization by regulating immune-related genes, which represent the downstream effectors activated by Toll pathways [60]. Peptidoglycan recognition proteins (PGRPs) detect Lys-type peptidoglycan (PG) from gram-positive bacteria, which leads to the activation of Toll signaling pathway, and ultimately increase the synthesis of an array of potent antimicrobial peptides by the fat body [61]. Previous studies have shown that the elevated expression levels for PGRPs are associated with *Varroa* infestations in larvae [26,39]. In this study, we found that the transcript level of a Peptidoglycan Recognition Protein (PGRPs) family member (GB47805) was up-regulated in *T*. *mercedesae*-infested bees. Our findings are in line with existing reports in which exposure to parasitic mites or arguably to viruses and other microbes carried by mites affect immunity traits in honey bees.

MRJPs are also a family of antimicrobial peptides that protect royal jelly from bacterial infection via forming short digestion products of MRJP. The mrjp 1 precursor (GB55205), MRJP5 (GB55208), MRJ6 (GB55207) were significantly up-regulated in the heads of infested honey bees that exhibited olfactory learning than those in control group (S3 Table). MRJP1 is an important multifunctional protein in the brain of honey bees. Honey bees with weak learning ability exhibited low expression levels of mrjp 1 in Kenyon cells in the mushroom bodies [62,63]. Moreover, neonicotinoid pesticides depressed the expression of MRJP genes in honey bees brains, thereby impaired olfactory learning in honey bees [64]. Our finding may provide further evidence for the role of MRJPs in the development of learning ability of honeybees. Ca^2+^/calmodulin-dependent protein kinase II (CaMKII) has been proposed as the integral component in mediating long-term memory formation that is specific for the learned odor in honeybees mushroom bodies [65]. Neurochondrin is a novel cytoplasmic protein that acts as a negative regulator of Ca^2+^/calmodulin-dependent protein kinase II (CaMKII) phosphorylation and is essential for the learning process in mammals [41]. We found that a gene encoding neurochondrin homolog protein (GB45484) was greatly downregulated (28.8-fold) in mite-infested honey bees that showed olfactory learning ability. Activation of CaMKII leads to enhancement of synaptic transmission and provides a tag to confer stimulus specificity as well as supporting natural odor preference learning [66]. Our results implied that the infested bees required higher CaMKII activity level in order to form learning memory. Obviously, whether the transcription level of neurochondrin is correlated to the protein level as well as the activity level needs to be proved in the future.

So far, the specific mechanism of action on olfactory performance remains unclear. In this study, we also analyzed the gene expression changes between bees with different sucrose responsiveness statuses. Previous studies have demonstrated a general down-regulation of protein-coding genes after associative olfactory learning in *A*. *mellifera* [67,68]. In this study, more down-regulated genes were identified in bees showing sucrose responsiveness in both CK and infested group, indicating that the activation of some related genes occurred in olfactory dysfunction (S4 Fig). We found that in both infested group and CK group, the vast majority of gene alterations occurred in the comparison between bee with and without PER, no matter having learning ability or not (S4 Fig). It implied that changes in overall gene expression in the honeybee brain was occurred after sucrose stimulation rather than memory formation. More DEGs were identified in the infested group than that in the CK group, which reflected the intensification of internal disorders and the insufficient resilience caused by the infestation. According to GO classification, the most significantly enriched term in both CK and infested group was associated with biological adhesion (GO:0022610) (Fig 6A and 6B). The cell adhesion molecule gene such as cadherin (GB47118, GB40703), neural-cadherin (GB45972, GB45970), protocadherin (GB53331, GB49977, GB51276) which play an important role in olfaction, were down-regulated in the bees with sucrose responsiveness (Fig 6C and S8 Table). Additionally, the cadherin-associated protein, catenin (GB44972) was also down-regulated in bees with normal olfactory sensory. Parker et al. have demonstrated that bees performing rapid hygiene express up-regulation of the Down syndrome cell adhesion molecule genes, which appeared to be involved in *Varroa* resistance [39]. The catenin-mediated cadherin adhesion may affect the targeting of olfactory sensory neuron by restricting axons to the outer olfactory nerve layer until they reach the appropriate domain of the olfactory bulb [69]. Here, we propose that modulation of cell adhesion molecule genes reflects their engagement in olfactory conditioning and PER of the bees. The abnormal olfactory performance in honeybees are possibly caused by the abnormal intracellular signal transduction of neuronal communication in electrical synapses via activation of cadherin systems, and needs further evidence for validation [70]. The odorant binding proteins (OBPs) and odorant receptors (ORs) identified in this study showed clear separation between bees with different sucrose responsiveness statuses, no differences were observed between infested group and control group (Fig 6D). Unexpectedly, the expression levels of most odor binding proteins and odorant receptors in bees with normal olfactory sensory were significantly lower than those of bees with olfactory dysfunction in both CK and infection groups (Fig 7A). Only a chemosensory protein 1 (GB43823) was up-regulated in bees with normal olfactory performance. The down-regulation of these candidate genes do not seem to support a higher olfactory sensitivity in bees with sucrose responsiveness. These results implied that the dysfunctional olfactory behaviors may require multiple molecular mechanisms underlying the olfactory recognition. Many studies have implied that changes in gene expression are not frequently correlated to the protein level [71]. It is difficult to establish a direct correlation between the differences in gene expression in transcriptomics and physiological functions. Whether the suppression of chemosensory related genes reported herein related to the sensitive olfactory sensation is questionable and requires further study.

The mushroom bodies (MB) are central brain neuropils that are fundamentally involved in learning and memory in bees [72]. Previous findings showing that hydroxyurea treatment induced ablation of MB by volume changes and by changes in protein expression, which are considered to play a role in synaptic plasticity, learning, and memory [73]. The ablation of MB neuropil may cause an increase in the volume of the lateral calyxin within the same brain side and thereby altered the arborization pattern of olfactory projection neurons [74]. We found that the mean thickness of mushroom bodies in infested honey bees was significantly thicker than that in non-infested honey bees (Fig 8B). The induction of precocious foraging in honey bees also accelerated the expansion of mushroom body neuropil volume [75]. We speculated that *T*. *mercedesae* infestation would promote early development in honey bees and that the rapid neuron growth would promote the thickening of the mushroom bodies in honey bees. The rapid growth of neurons also accelerates apoptosis in the neuronal system, which is an important reason for the abnormal sugar sensitivity of infested honey bees. Further studies are required to confirm the identified morphological alterations in mushroom body involved in proximate learning mechanisms underlying the defense response to *T*. *mercedesae* parasitization.

The antenna are the main chemosensory detection organs in honey bees. The antennal lobes contain the primary olfactory neuropil and are connected to the promotor area in the protocerebrum and to the mushroom bodies [76]. Letzkus et al. demonstrated that the right antenna of honey bees responds better to odor learning than the left antenna [34]; additionally, the mean number of sensilla placodea is significantly higher in the right antenna than in the left antenna [77]. In this study, we tested the number and size of olfactory cells in the 4th, 7th, and 8th right antennae (Fig 7A–7C). The data showed that *T*. *mercedesae* infestation did not affect number or sensilla placodea cell size of the right antennae, suggesting that observed learning and memory impairment in this study may be not related to the antenna morphology.

Altogether, those findings provide novel insights into the response of host honey bees to *T*. *mercedesae* infection. Our data highlight a series of behavioral changes associated with *T*. *mercedesae* infection in adult honey bees. In this study, we found that *T*. *mercedesae* infestation affected the olfactory performance of honeybees, which is potentially caused by changes in gene expression involved in immune systems, carbohydrate transport and metabolism, and regulation of CaMKII activity. In addition, we found genes function in cell adhesion play an essential role in olfactory sensory in honey bees. Whether the transcription level of these genes is correlated to the protein level needs to be proved in the future. Further studies are required to confirm the identified responsive genes involved in proximate mechanisms underlying the resistance to *T*. *mercedesae*.

## Supporting information

S1 Fig The flight mill used to test the flight ability of tethered forager bees. (TIF)

S2 Fig *Apis mellifera* infested with *Tropilaelaps mercedesae* (untreated control) used for the PER paradigm. (TIF)

S3 Fig The survival rate of bees after homing ability test. (TIF)

S4 Fig The differentially expressed genes (DEGs) between honey bees with different sucrose responsiveness in control group or infested group. **A.** Histogram of the number of DEGs identified in various comparisons. **B.** Venn diagram of up-regulated DEGs in various comparisons. **C.** Venn diagram of down-regulated DEGs in various comparisons. DEGs indicate that these genes were expressed more or less in latter group group than in the corresponding former group.
(TIF)

S1 Table Summary of the Illumina transcriptome assembly for samples tested in this study. (XLSX)

S2 Table Information of DEGs between mite-infested and non-mite infested honeybees associated with different sucrose responsiveness and learning statuses. (XLSX)

S3 Table DEGS are abundant in Carbohydrate transport and metabolism in comparison CKL vsTL. (XLSX)

S4 Table Summary of DEGS identified in comparison of CKSN vs. CKL and TSN vs. TL. (XLSX)

S5 Table GO classification of theDEGs identified in comparison CKSN vs. CKL. (XLSX)

S6 Table GO classification of theDEGs identified in comparison TSN vs. TL. (XLSX)

S7 Table DEGS that assigned as chemosensory genes identified in various comparisons. (XLSX)

S8 Table DEGS that associated to bioadhesion identified in various comparisons. (XLSX)
