# Supplementary figures and images for: Tropilaelaps mercedesae parasitism changes behavior and gene expression in honey bee workers
Source: PLoS Pathog. 2021 Jul 8;17(7):e1009684. doi: 10.1371/journal.ppat.1009684 (PMC8266070; doi:10.1371/journal.ppat.1009684)

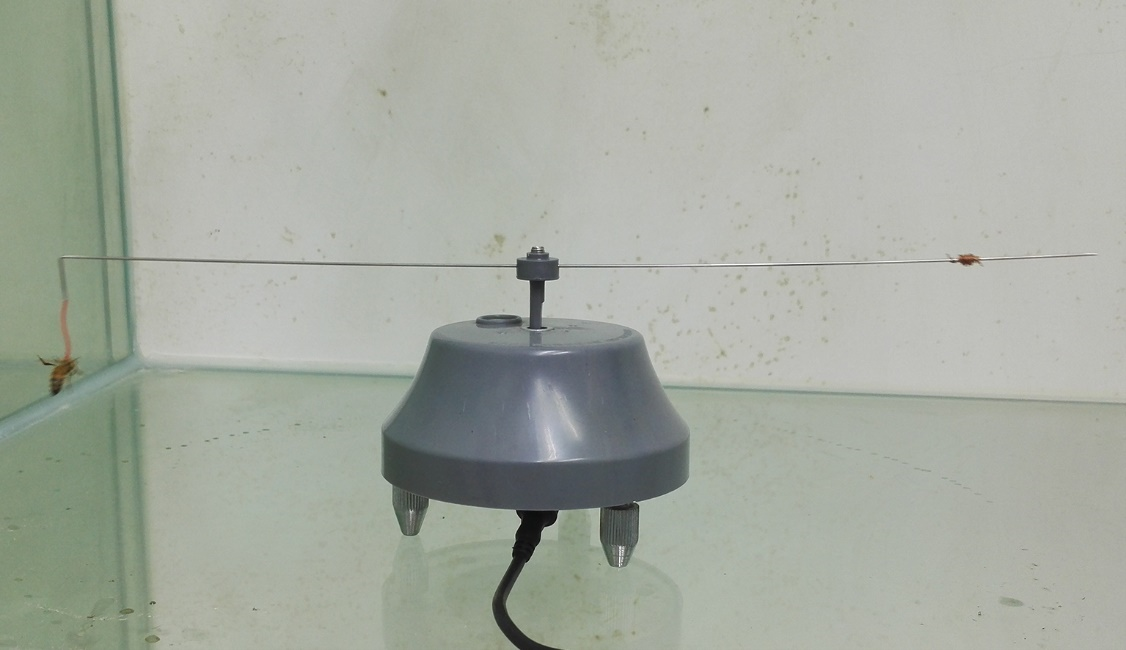

Supplement: S1 Fig — (TIF) [file ppat.1009684.s001.tif]

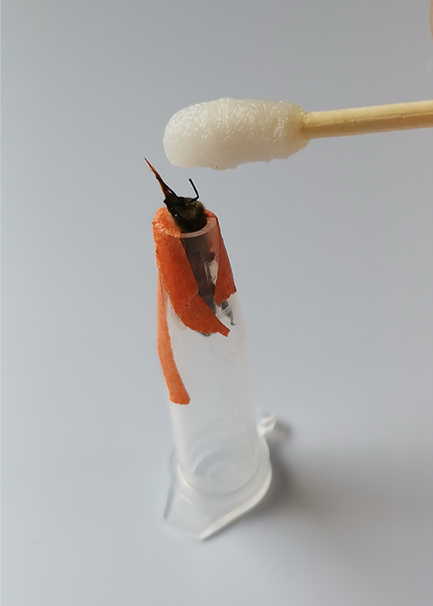

Supplement: S2 Fig — (TIF) [file ppat.1009684.s002.tif]

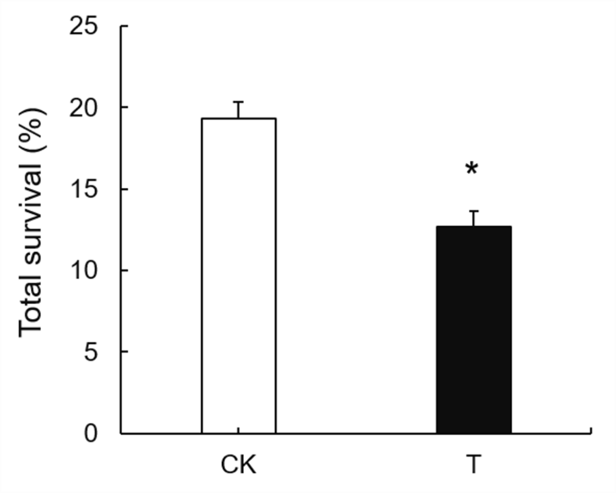

Supplement: S3 Fig — (TIF) [file ppat.1009684.s003.tif]

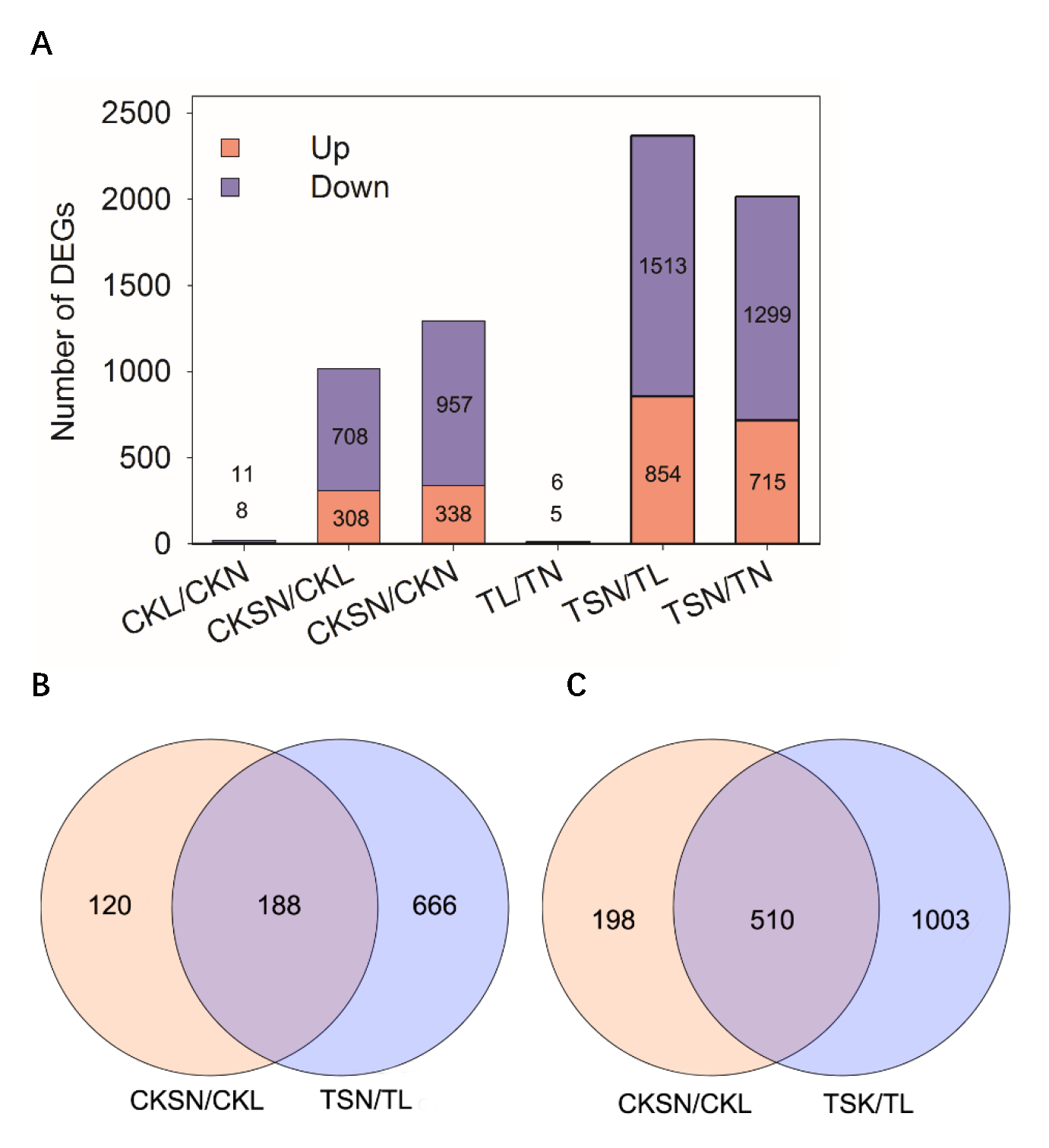

Supplement: S4 Fig — A. Histogram of the number of DEGs identified in various comparisons. B. Venn diagram of up-regulated DEGs in various comparisons. C. Venn diagram of down-regulated DEGs in various comparisons. DEGs indicate that these genes were expressed more or less in latter group group than in the corresponding former group. (TIF) [file ppat.1009684.s004.tif]
